# The association of ultrasound assessment of gallbladder wall thickness with dengue fever severity

**DOI:** 10.1186/s13089-022-00262-w

**Published:** 2022-03-24

**Authors:** Mohd Anwar Ibrahim, Siti Suhaila Hamzah, Julina Md Noor, Mohamad Iqhbal Kunji Mohamad, Mohd Fazrul Mokhtar, Mohamad Rodi Isa, Mohammed Fauzi Abdul Rani

**Affiliations:** 1 Department of Emergency and Trauma, Hospital Sungai Buloh, Sungai Buloh, Malaysia; 2 Emergency Department, Faculty of Medicine, Universiti Teknologi MARA, Sungai Buloh, Malaysia; 3 Public Health and Preventative Medicine, Faculty of Medicine, Universiti Teknologi MARA, Sungai Buloh, Malaysia; 4 Department of Internal Medicine, Faculty of Medicine, Universiti Teknologi MARA, Sungai Buloh, Malaysia

**Keywords:** Severe dengue, Gallbladder, Ultrasonography, Emergency, Critical care, Risk assessment

## Abstract

**Objectives:**

To evaluate the association between ultrasound assessment of gallbladder wall thickness (GBWT) among severe dengue patients and dengue patients with warning signs to their clinical outcomes.

**Methods:**

A prospective, cross-sectional study involving adult dengue patients presented to our emergency department between March until September 2018. The patients were classified based on WHO classification. A gallbladder wall scan was performed on all patients.

**Results:**

A total of 44 patients were enrolled into the study; majority of the patients with GBWT had severe dengue, significantly more than the dengue patients with warning signs (90.5% sensitivity; 69.6% specificity). The sensitivity of GBWT in determining admission to critical care areas or general ward was 100% with a specificity of 62.1%. Our analysis showed that the two variables significant in determining the severity of dengue were age (*p* = 0.045) and GBWT (*p* < 0.001). Both factors together gave 81.0% sensitivity and 78.3% specificity in predicting patients for severe dengue. The receiver operator characteristic curve revealed that using variable GBWT status can discriminate 87.1% (95%CI 66.3, 93.7%) of having severe dengue or dengue with warning signs.

**Conclusion:**

The finding of GBWT when consolidated with other clinical parameters may assist clinicians to perform risk stratification in the emergency department and become another adjunct to the assessment of severe dengue.

## Introduction

Over the last decade, the global prevalence of dengue has risen significantly. It has become the most common arthropod-borne viral disease which carries high morbidity and mortality. The incidence in Malaysia has continued to rise from 19,884 cases in 2011 to 13,0101 cases in 2019. This is associated with increased mortality, from 147 deaths in 2018 to 182 deaths in 2019 [1]^.^

Dengue has a broad spectrum of illnesses ranging from being asymptomatic to developing high-grade fever with or without warning signs of dengue and even severe disease associated with plasma leakage. The mechanism that caused individuals to progress to severe dengue is poorly understood, and the study of its predictors is rather complex [2]. Currently, there are no routine laboratory biomarkers to predict the severity of dengue infection or monitor the effectiveness of standard management [3].

During an upsurge of dengue cases, a rapid bedside investigation would be invaluable. The ability to perform risk stratification early in the patient’s course of illness may assist physicians in deciding how intensively the patient should be observed and treated. A focused ultrasound assessment among patients with warning signs, for example, can be used to look for those who are at risk of developing severe dengue or plasma leakage [4]. This has also been shown to coincide with clinical improvement whereby resolving ultrasound findings were found at the time of discharge [5]. These sonographic features include gallbladder wall thickening (GBWT), pleural effusion, ascites, hepatomegaly, and splenomegaly [6]. Among these, GBWT was found to be the most common [7–9] and most specific in severe dengue patients [5].

Despite the increasing prevalence of dengue in Malaysia which causes more deaths, there has never been a local study looking at GBWT and outcome. Therefore, our study aims to look at the association between GBWT among severe dengue patients and patients with warning signs from our center and its relevance to their outcomes.

## Methodology

This study was conducted at Hospital Sungai Buloh (HSB), Malaysia, a large suburban tertiary care hospital with a total of 160,000 emergency attendance annually. The study was prospective and cross-sectional in nature with convenience sampling. All scans were performed in the Emergency Department (ED) of HSB by the principal investigator who has completed a national ultrasound fellowship in point-of-care ultrasound. This study received ethical approval from the Malaysian Medical Research and Ethics Committee (MREC) and registered with National Medical Research Registry (NMRR).

### Sample size

The method used to calculate sample size indicates that the proportion of the difference in gallbladder wall more than 0.3 cm in severe dengue group was 0.965, whereas the proportion in dengue with warning signs group was 0.576 [7]. With an additional 10% dropout rate, the sample size is 20 samples per group (severe dengue and dengue with warning signs).

### Patient selection

From March to September 2018, all adult dengue patients treated for dengue fever with warning signs or severe dengue were selected for this study. The diagnosis of dengue was made using point-of-care Dengue Combo Kit test, with a positive NS1-antigen, or positive dengue IgM. Based on the World Health Organization (WHO) 2009 definitions of dengue, the patients were then grouped into (i) dengue with warning signs, (ii) severe dengue.

All patients who consented for the study were recruited in the emergency department and communicated in either Malay or English. Patients with pre-existing gallbladder disease were excluded from this study and the written consent was obtained before the ultrasound examination.

The principal investigator performed a gallbladder ultrasound examination on all patients enrolled. The investigator was not involved with the management of these patients. The in-hospital physicians treating the patients were also blinded to the findings of the ultrasound. All patients were followed up until discharge to identify the details of the admission, the length of hospital stay, and in-hospital mortality.

### Gallbladder ultrasound examination

GE Logiq P9 ultrasound machine with a convex probe 4C-SC 5 MHz was used. The scan was performed with patients lying in a supine position, using the intercostal approach with the probe placed over the right hypochondrium region. GBWT was measured at the anterior part of the gallbladder. GBWT < 0.3 cm is considered normal. The pattern of the gallbladder wall was also described.

###  Data analysis

Statistical analysis was carried out using SPSS 25.0 IBM (SPSS Inc, Chicago, IL, US). The descriptive statistics were presented using mean and standard deviation for a continuous variable, while the absolute number and percentage for the categorical variable. The difference in the length of stay with GBWT was analyzed using an independent t-test. The difference of GBWT with the severity of dengue and admission toward and critical care areas was analyzed using a Chi-square test. The predictors for severe dengue were analyzed using univariate and multivariable logistic regression. The discrimination of having severe or not severe dengue was determined by calculating and plotting the receiver operative characterization (ROC) curve. All reported *p*-values are two-sided and deemed statistically significant at *α* = 0.05.

## Results

From March to September 2018, 44 patients were enrolled in the study. All cases were confirmed as dengue with NS1 and IgM tests done during the presentation. According to the definition by WHO, the phases of illness were equally divided into febrile and critical phases.

**Fig. 1:**
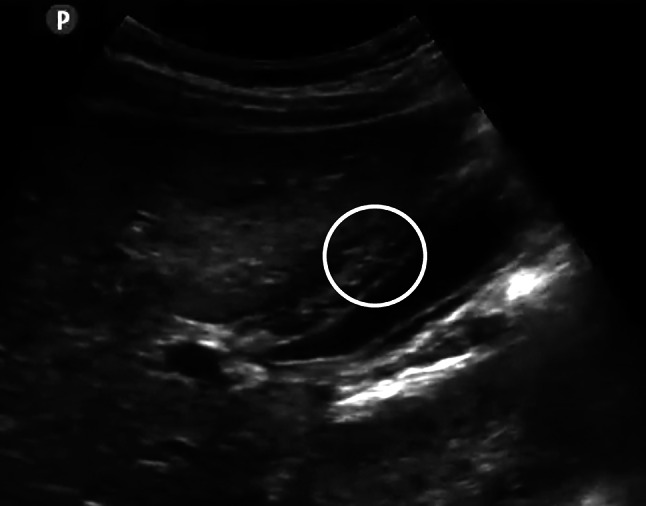
The gallbladder wall is thickened, appearing as though the wall has separated looks with striated structure in between

Figure 1 is a typical GBWT found in a dengue patient. The wall appears to have two layers with striated or reticular pattern in between.

The variable assessed including the severity of dengue, admission, gallbladder wall thickening (GBWT), and length of stay. Table 1 shows the clinical characteristics of the patients. The majority of patients with GBWT were in severe dengue (73%), and those with severe dengue mainly were admitted to the high dependency ward (HDW)/intensive care unit (ICU) (66.7%). The mean length of stay (LOS) was also relatively longer in the severe dengue group [mean 4.43 days (1.12)].

**Table 1:** Baseline clinical characteristic of 44 patients

Characteristics	Dengue with warning signs (*n* = 23)	Severe dengue (*n* = 21)	All cases
Age, mean	41 ± 15	36 ± 15	39 ± 15
Day of illness during presentation, mean	5.04 ± 1.89	4.29 ± 1.35	4.68 ± 1.68
Phase			
Febrile	15 (65.2%)	7 (33.3%)	22 (50%)
Critical	8 (34.8%)	14 (66.7%)	22 (50%)
GBWT			
Yes	7 (30.4%)	19 (90.5%)	26 (59.1%)
No	16 (69.6%)	2 (9.5%)	18 (40.9%)
Admission			
General ward	22 (95.7%)	7 (33.3%)	29 (65.9%)
HDW/ICU	1 (4.3%)	14 (66.7%)	15 (34.1%)
Length of stay, mean	3.83 ± 2.50	4.43 ± 1.12	4.11 ± 1.97

On further analysis, there was a significant difference in GBWT between severe dengue and dengue with warning signs (*p* < 0.001) with 90.5% sensitivity and 69.6% specificity, as shown in Table 2. The admission to a general ward and HDW/ICU were also significantly different with GBWT (*p* < 0.001). The sensitivity of GBWT in determining admission to ICU/HDW or general ward is 100% with a specificity of 62.1%. However, there was no significant difference in the length of stay with GBWT (*p* = 0.38).

**Table 2:** The significance of GBWT in relation to dengue severity and admission type

Variable	GBWT		*P* value	OR (95%CI)
	Yes (*n* = 26)	No (*n* = 18)		
Severity			< 0.001	21.7 (3.9, 119.6)
Severe dengue	19 (73.1%)	2 (11.1%)		
Dengue with warning signs	7 (26.9%)	16 (88.9%)		
Admission			< 0.001	–
HDW/ICU	15 (57.7%)	0 (0.0%)		
General ward	11 (42.3%)	18 (100.0%)		

Table 3 demonstrates that using univariate logistic regression to analyze variables that are more prevalence in severe dengue, the GBWT and admission to HDW/ICU were significantly higher in prevalence with both *P* < 0.001, in contrast with the phase of dengue fever.

**Table 3:** Predictors of severe dengue as assessed by univariate logistic regression

Variable	Dengue with warning signs	Severe dengue	*P* value
Age, mean	41 ± 15.3	35.6 ± 15.1	0.244
Day of illness during presentation, mean	5.04 ± 1.89	4.29 ± 1.35	0.147
Phase			0.038
Febrile	15 (65.2%)	7 (33.3%)	
Critical	8 (34.8%)	14 (66.7%)	
GBWT			< 0.001
Yes	7 (30.4%)	19 (90.5%)	
No	16 (69.6%)	2 (9.5%)	
Admission			0.001
General ward	22 (95.7%)	7 (33.3%)	
HDW/ICU	1 (4.3%)	14 (66.7%)	
Length of stay, mean	3.83 ± 2.50	4.43 ± 1.12	0.333

With multivariable analysis as shown in Table 4, there were two variables found significant in determining the severity of dengue, i.e., age (*p* = 0.045) and GBWT (*p* < 0.001). Combining both factors bring 81.0% sensitivity and 78.3% specificity of predicting someone with severe dengue.

**Table 4:** Predictors of severe dengue using multivariate logistic regression

Variable	Adjusted odds ratio	95% CI	*P* value
Age, mean	1.05	1.01–1.07	0.045
GBWT, Yes	33.12	4.82–27.62	< 0.001

**Fig. 2:**
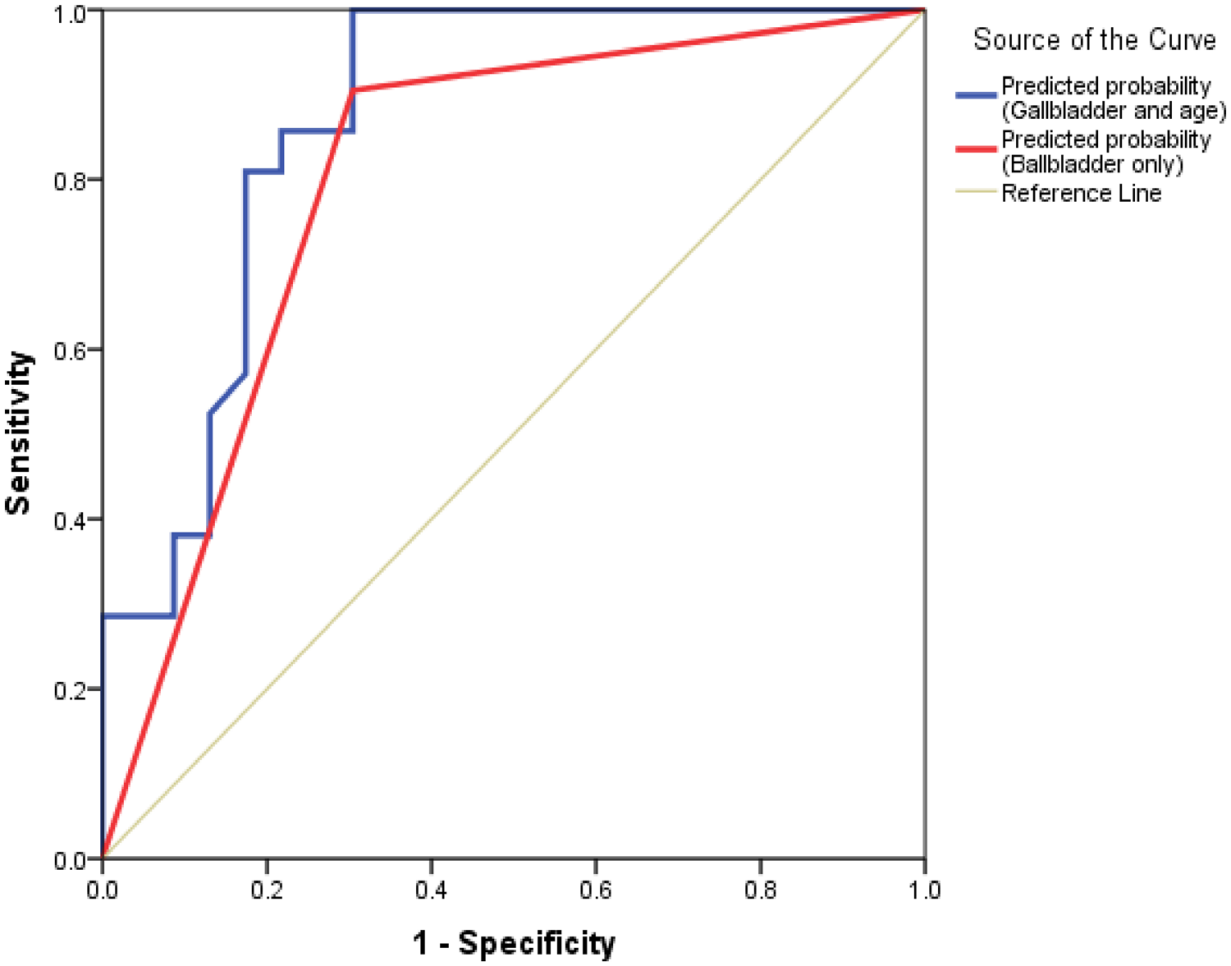
ROC curves for GBWT and age

The receiver operator characteristic (ROC) curve in Fig. 2 revealed that using variable GBWT status only can discriminate 87.1% (95% CI 66.3, 93.7%) of having severe dengue or dengue with warning signs. However, by combining GBWT and age, it can discriminate 87.1% but with a narrower confidence interval (95% CI 76.3, 97.9%) of having severe dengue.

## Discussion

The changes in dengue fever such as generalized vascular damage increased permeability of blood vessels to plasma protein, and effusion in the pericardial, pleural, and peritoneal cavity was first described by Bhamarapravati et al. in their retrospective analysis of autopsy findings [10]. Wang et al., later on, identified these findings through ultrasound with the addition of thickened gallbladder in dengue. In our study, the overall frequency of thickened gallbladder walls was 59.1%. Other studies have quoted the incidence ranging from 32% to as high as 89.2% [11–14]. There was no mortality reported in our patients. We found a difference in length of hospital stay, but this difference was not statistically significant.

Our study showed that GBWT is sensitive in predicting severe dengue with a high sensitivity of 90.5%. To our knowledge, this is the first study written from a Malaysian sample. A gallbladder wall of more than 0.3 cm is taken as the cut-off point. Although thickened gallbladder wall is not specific and is associated with other conditions [15], it has to be taken into clinical context. Studies over the last 3 decades have consistently proven the association of GBWT and severity of dengue [13, 16–18]. Kim et al. reported that dengue patients with signs of fluid leakage were higher in the patients with GBWT than those without GBWT [19]. This finding is important as GBWT is likely to be one of the few findings that cannot be identified through clinical examination. The introduction of dengue with warning signs in WHO 2009 guideline stated that all patients with warning signs require admission. However, it was found that no single warning signs can predict disease progression [20]. GBWT can therefore become another adjunct to the assessment of severe dengue.

GBWT also showed a significant difference in determining admission either to the general ward or critical care areas. This study demonstrated that all the patients admitted to critical care areas had GBWT, leading to a sensitivity of 100%. However, there were patients with GBWT that were admitted to a general ward which explains the low specificity of GBWT. This is almost similar to the findings by Setiawan et al., whereby a sonographic finding of GBWT ≥ 0.3 cm had 93.8% sensitivity to indicate the need for admission and monitoring, while ≥ 0.5 cm had 91.7% specificity in identifying dengue patient who is at risk of developing hypovolemic shock [21]. In addition to that, we were able to build ROC curves, as shown in Fig. 2, to predict severe dengue based on GBWT alone. The combination of GBWT and age increased the sensitivity of predicting severe dengue. Putting into context the clinical findings, hematocrit level and the addition of a specific sonographic finding among dengue patients can be a predictive tool for early selection of patients to critical care area.

As with many ultrasound studies, the sample number is small. However, we did calculate the sample size needed to reach a meaningful number. This study was performed in ED, to be pragmatic and assist doctors in ED. Hence there was no ultrasound follow-up study to determine if the dengue patients with warning signs developed thickening of the gallbladder wall later on in the ward. There was also no repeated ultrasound at discharge to look for resolution of GBWT.

## Conclusion

Our findings affirm a previous study that shows an association between GBWT and the severity of dengue. GBWT and age can predict the severity of dengue with high sensitivity. When consolidated with clinical presentations, this finding may assist clinicians in further management of severe dengue.

## Dissemination of results

Results from this study were shared with the staff members at the data collection site and Universiti Teknologi MARA through formal presentations by the authors.

## Data Availability

All data and materials are kept by the first and corresponding authors.
